# Dimensionality, reliability, invariance, and item analysis of the depression, anxiety, and stress scale-21 (DASS-21) in Honduran and Colombian university students

**DOI:** 10.1186/s40359-025-03742-5

**Published:** 2025-11-25

**Authors:** Marcio Alexander Castillo-Díaz, Carlos Alberto Henao Periañez

**Affiliations:** 1 Departamento de Psicología/ Maestría en Psicometría y Evaluación Educativa, Facultad de Ciencias Sociales, Universidad Nacional Autónoma de Honduras, Tegucigalpa, Honduras; 2 Escuela de Enfermería, Facultad de Salud, Universidad del Valle, Cali , Colombia

**Keywords:** DASS-21, Structural validity, Graded response model, Differential item functioning, University students

## Abstract

**Background:**

The 21-item Depression, Anxiety, and Stress Scale (DASS-21) is a widely used instrument for assessing psychological distress in diverse populations worldwide. Nonetheless, in the Honduran and Colombian contexts there is a paucity of studies evaluating the scale’s factorial structure, examining item-level parameters, and investigating measurement invariance at both the dimension and item levels. The present study analyzes the psychometric properties—structural validity, reliability, measurement invariance, item-level characteristics, and differential item functioning—of the DASS-21 among university students in Honduras and Colombia.

**Methods:**

A cross-sectional design was employed, involving 2,271 university students (1,112 from Honduras and 1,159 from Colombia). The full sample comprised 68.10% women, with a mean age of 22.21 ± 5.03 years. The participants completed the DASS-21 through an online form. As part of the factorial modeling, several structures were tested via confirmatory factor analysis; configural, metric, and scalar invariance across countries were examined; and the reliability of the best-fitting model was estimated. In addition, an item response theory evaluation was carried out with a multidimensional graded response model (MGRM) that estimated two parameters—discrimination and thresholds—and differential item functioning (DIF) was assessed.

**Results:**

The correlated three-factor model of the DASS-21 was identified as the best-fitting structure, as indicated by satisfactory fit indices (comparative fit index [CFI] and Tucker–Lewis Index [TLI] > .95; root mean square error of approximation [RMSEA] < .08) and factor loadings (λ > .40; *p* < .01). Acceptable reliability coefficients were obtained for each dimension (Ω > .80). This model demonstrated configural, metric, and scalar invariance across countries. Findings from the multidimensional graded response model showed acceptable discrimination and threshold estimates for all the items. Uniform differential item functioning was detected in four items of the scale (*p* < .05).

**Conclusions:**

The results provide favorable psychometric evidence supporting the use of the DASS-21 as a screening tool for assessing depression, anxiety, and stress among Honduran and Colombian university students. Evidence of measurement invariance allows cross-cultural comparisons of latent means. Nevertheless, caution is advised when making country-specific item-level comparisons for the items that exhibited DIF.

**Supplementary Information:**

The online version contains supplementary material available at 10.1186/s40359-025-03742-5.

## Background

Mental health is a fundamental component of human well-being and social development, significantly influencing quality of life and interpersonal relationships [1]. Mental disorders—such as depression, anxiety, and stress—pose a major challenge for public health systems worldwide. These conditions rank among the leading causes of years lived with disability and adversely affect the quality of life of millions of people, irrespective of their sociocultural context [2]. This situation is particularly critical in low- and middle-income countries, where the prevalence of depressive and anxiety disorders is higher compared to other regions, and where the structural limitations of health systems exacerbate the negative impact on university students and other vulnerable populations [3, 4].

Among high-risk population groups, university students constitute one of the populations most vulnerable to mental disorders because they are exposed to various stressors [5–7]. These stressors include academic pressure, the challenges inherent in the transition to adulthood, and the uncertainty associated with their future professional prospects [8, 9]. Numerous studies have documented a high prevalence of mental health-related symptoms in this population group, a situation that negatively affects both their academic performance and overall well-being [10–13].

Early identification of depression, anxiety, and stress symptoms requires the use of valid and culturally appropriate assessment tools [10]. The 21-item Depression, Anxiety, and Stress Scale (DASS-21) is a brief self-report instrument that has been widely used internationally to measure these psychological constructs [14, 15]. The scale is grounded in the tripartite theoretical model of anxiety and depression [16]. The psychometric properties of the DASS-21 have been analyzed across multiple languages and contexts [17–20].

With respect to the dimensionality of the scale, systematic review and meta-analytic evidence indicated that both exploratory and confirmatory approaches have been employed, with confirmatory factor analysis (CFA) being the most frequently applied [21]. While the original three-factor structure has received empirical support [18, 22, 23], some CFA studies have reported inadequate fit indices and high interfactor correlations [17, 19]. On the other hand, research has evaluated alternative solutions, such as unidimensional, hierarchical, and bifactor models, with the latter generally providing the best fit [20, 21, 24]. In addition, although less frequently documented, a study reported residual correlations among items, indicating some overlap in content and the potential need to model residual covariances to improve fit [23]. Overall, these findings highlight the complexity of the scale’s dimensionality and the variability of results across studies.

Specialized literature concurs that the instrument’s validity evidence cannot be assumed to be generalizable universally; rather, it must be empirically evaluated so that it is sensitive to the specific sociocultural context in which it is analyzed [25–27]. Systematic reviews indicate that further investigation of the scale’s measurement invariance is warranted, considering sociodemographic and cultural variables that may affect item scores in particular settings [19]. It is also necessary to evaluate the scale’s factorial structure, reliability, and item-level parameters to enhance the interpretation and applicability of findings across diverse sociocultural settings [14].

Several recent studies have evaluated the psychometric properties of the DASS-21 in educational contexts across Asia, Africa, and Europe, confirming its utility as a measurement tool [25, 26, 28–30]. Systematic literature reviews indicate that most research on the DASS-21 has focused on analyzing the scale’s latent factorial structure [14, 21]; however, few studies have incorporated contemporary item response theory (IRT) analyses in evaluating the measure [22, 31].

Analyses of different factorial structures provide information about the latent dimensions underlying the items and their overall fit [32]. In turn, incorporating IRT-based analyses can yield valuable insights into each item’s performance by examining its discriminative capacity for identifying different levels of the assessed dimension and by locating each item along the latent trait continuum, determining the segment in which it contributes the most information to the assessment [33]. Such information is especially important for implementing individualized intervention approaches aimed at identifying and reducing specific symptoms.

In Latin America—particularly in countries such as Honduras and Colombia—there is a paucity of research examining the structural validity, reliability, measurement invariance, and item-level characteristics of the DASS-21. In Honduras, the scale has been employed in prior studies [34, 35]; however, those investigations did not evaluate alternative factorial models that might better capture the scale’s dimensionality in this context in depth, nor did they conduct item-level parameter analyses.

On the other hand, to our knowledge, only one published study has addressed the factorial structure of the DASS-21 in Colombia [24]. This study assessed internal consistency solely through Cronbach’s alpha, even though contemporary psychometric literature has noted important limitations of alpha as a reliability index for psychological constructs [36, 37]. Evidence for DASS-21 based on IRT also remains scarce in Colombia.

The focus on Honduras and Colombia is particularly relevant because, as countries from Latin America, they share cultural and linguistic characteristics but differ in terms of socioeconomic indicators [38], health system infrastructure [39], and higher education environments [40]. For instance, according to the Human Development Report 2025, Colombia has a Human Development Index (HDI) of 0.788 (high), whereas Honduras has an HDI of 0.645 (medium), underscoring disparities in socioeconomic development [38]. Examining measurement invariance across these contexts allows for testing whether the DASS-21 functions equivalently in populations that are regionally and linguistically similar but exposed to different structural conditions, thereby strengthening its cross-cultural applicability in Latin America.

The lack of psychometric studies on the DASS-21 in Honduras and Colombia hinders efforts to capture cultural nuances that the scale’s latent variables may display in these settings. In this context, the absence of robust evidence limits the use of the instrument as a screening tool that could support evidence-based mental-health programs in university environments. Furthermore, factorial- and item-level invariance evidence across countries is essential for conducting sound cross-cultural analyses and making appropriate score comparisons [41].

### The current study

Considering the above arguments, the present study aims to examine the psychometric properties—structural validity, reliability, measurement invariance, item‒parameter estimates, and differential item functioning—of the DASS-21 among university students in Honduras and Colombia. Validating the scale locally is essential not only for ensuring diagnostic accuracy but also for strengthening institutional responses to mental-health challenges in academic settings. Moreover, integrating factorial modeling with IRT analyses can increase the precision, fairness, and practical validity of scores in the contexts in which they are applied [42, 43]. Together, these analyses yield information about the scale’s overall latent structure and the specificity of each item, strengthening the empirical evidence and culturally sensitive use of the DASS-21 in Latin American university students.

## Method

### Study design and participants

This cross-sectional study included two samples of university students from Latin America—Honduras and Colombia. Eligibility criteria required active enrollment and an age of at least 18 years. Using nonprobability convenience sampling, the study included 2,271 students: 1,112 Hondurans (49%) and 1,159 Colombians (51%). Table 1 summarizes the sample’s sociodemographic characteristics.

**Table 1:** Sociodemographic characteristics of the sample

Variables	Honduras *n* = 1112	Colombia *n* = 1159	Full sample *n* = 2271
*Age (Mean* ± *SD)*	22.64 ± 5.36	21.80 ± 4.67	22.21 ± 5.03
*Sex, n (%)*			
Women	741 (66.64%)	828 (71.44%)	1569 (68.09%)
Men	366 (32.91%)	329 (28.39%)	695 (30.60%)
Nonbinary	5 (0.45%)	2 (0.17%)	7 (0.31%)
*Marital status, n (%)*			
Single	997 (89.65%)	1077 (92.92%)	2074 (91.33%)
Married or common-law union	122 (10.07%)	79 (6.82%)	196 (8.41%)
Separated, divorced, or widowed	3 (0.28%)	3 (0.26%)	6 (0.26%)
*Field of study, n (%)*			
Humanities and social sciences	207 (18.67%)	223 (19.24%)	430 (18.96%)
Engineering	213 (19.21%)	109 (9.41%)	322 (14.20%)
Economics and business sciences	258 (26.26%)	222 (19.16%)	480 (21.16%)
Health sciences	222 (20.02%)	440 (37.96%)	662 (29.19%)
Law and political sciences	89 (8.02%)	130 (11.22%)	219 (9.66%)
Natural and exact sciences	123 (10.82%)	35 (3.02%)	155 (6.83%)
*Area of residence, n (%)*			
Rural	235 (21.13%)	124 (10.70%)	359 (15.81%)
Urban	877 (78.87%)	1035 (89.30%)	1912 (84.19%)

### Instrument

Depression, Anxiety, and Stress Scale–21 (DASS-21). It is a 21-item self-report measure that assesses psychological distress through symptomatology derived from the tripartite model of depression, anxiety, and stress [15, 44]. The present study used the Spanish version of the scale [45], which has previously been applied in Honduran [34] and Colombian samples [24]. Each dimension comprises seven items—depression (e.g., “I couldn’t get myself interested in anything”), anxiety (e.g., “I felt my hands tremble”), and stress (e.g., “I found it hard to wind down”). The respondents indicate how they have felt during the past two weeks on a four-point Likert scale ranging from 0 (never) to 3 (most or all the time). Higher scores reflect greater severity of the latent variables assessed.

### Procedures

The instrument and sociodemographic form were administered online through an electronic questionnaire created for this purpose. In Honduras, the survey was distributed to students at the country’s main public university and circulated nationwide across its regional campuses. The Colombian sample was distributed at two private universities in Cali, Valle del Cauca. Although nonprobability convenience sampling was used, the questionnaire was widely shared through institutional communication channels with students from all faculties and degree programs in both contexts, thereby maximizing reach and fostering sample heterogeneity.

This research strictly adhered to the ethical principles of the Declaration of Helsinki (and its subsequent amendments) and was conducted in accordance with national and institutional regulations. The project received institutional approval from the Vice-Rectory of Student Affairs of the National Autonomous University of Honduras (Oficio VOAE 205–2020), and endorsement from the Research Ethics Committee of Universidad Libre in Colombia (Ref. 20.09.20).

Data were collected from March to September 2021 in Colombia and from May to August 2021 in Honduras. All the students in the sample were informed about the study’s purpose, methodology, and potential risks and benefits. Participation was voluntary and contingent upon the participants signing an electronically informed consent form stating they could withdraw from the study any time. In both the Honduran and the Colombian surveys, the end of the questionnaire provided information on institutional psychological counseling services and contact details that students could access for individualized assistance.

### Data analysis

Data analysis proceeded in two phases: the first involved factorial modeling of the scale, and the second focused on an evaluation based on IRT. All analyses were conducted using R software (version 4.4.2) [46]. Factorial modeling was performed with the *lavaan* package (version 0.6–16) [47], supported by the *semTools* package (version 0.5–6) [48]. The IRT analyses were conducted with the *mirt* package, version 1.45.1 [49]. Prior to analysis, the data were inspected for missing values, and none were found. Descriptive statistics and category percentages for each DASS-21 item by country and full sample are presented in Tables S1 and S2 of the supplementary material.

**Fig.1:**
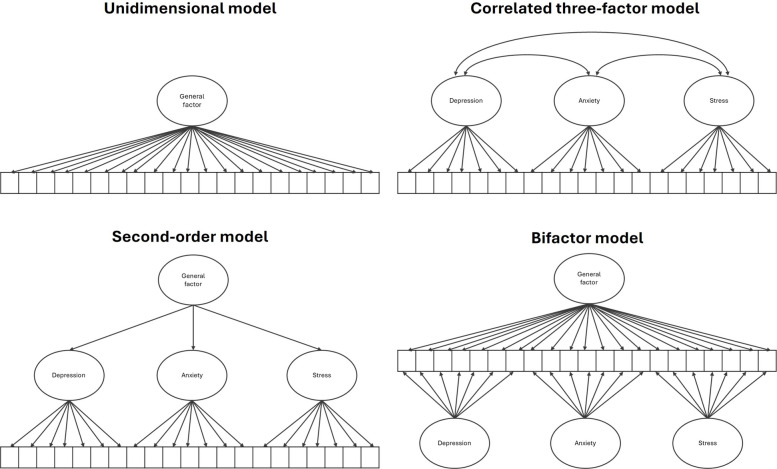
Factorial structures tested for the DASS-21

As part of the first phase, we conducted item-level confirmatory factor analysis (CFA), estimated reliability, and tested measurement invariance. CFA was performed to evaluate validity evidence based on the scale’s internal structure. Four factorial models previously documented for the DASS-21 [19] were tested: a) a unidimensional model—a single factor accounts for the variance of all 21 items, reflecting a unitary conceptualization of psychological distress; b) a correlated three-factor model—three oblique latent variables (depression, anxiety, stress) each explain the variance of their respective seven items; c) a second-order model—the same three first-order factors are retained, with a second-order general factor accounting for their covariance; and d) a bifactor model—a general factor directly explains the variance of all 21 items, alongside three orthogonal specific factors that each explain seven items. Figure 1 depicts the graphical representations of the tested models.

Model fit was evaluated separately for the Honduran, Colombian, and full samples. Given the ordinal nature of the items, we used the weighted least squares mean- and variance-adjusted estimator (WLSMV), which is appropriate for categorical variables [32]. Model fit was assessed with the comparative fit index (CFI), Tucker–Lewis index (TLI), and root mean square error of approximation (RMSEA). Values of CFI and TLI > 0.90 and RMSEA < 0.08 indicate acceptable fit [50]; more stringent thresholds of CFI and TLI > 0.95 and RMSEA < 0.06 denote good fit [51]. The best-fitting model was selected on the basis of acceptable fit indices and statistically significant factor loadings (*p* < 0.05) with λ ≥ 0.40, following the procedure used in prior research [52].

After the CFA, we estimated the reliability of the model that best represented the DASS-21 factorial structure. Internal consistency was assessed with McDonald’s omega and the composite reliability index [53]. Values above 0.70 were considered indicators of reliability [54]. The literature indicates that these coefficients are most appropriate for evaluating psychological constructs [36, 37]. As an additional indicator of reliability, we also reported the Average Variance Extracted (AVE) [55]. According to the literature, AVE values above 0.50 are considered acceptable [54].

We subsequently conducted a measurement invariance analysis using the model that showed the best fit indices and factor loadings in the CFA stage. This analysis evaluated whether the scale’s factorial structure was equivalent across nationalities. A multigroup confirmatory factor analysis (MGCFA) tested configural, metric, and scalar invariance, following established methodological and reporting conventions [56]. Invariance levels were examined sequentially by comparing nested models that imposed progressively stricter constraints [57].

Configural invariance assumes the same factorial structure in each group; metric invariance tests the equivalence of factor loadings; and scalar invariance adds the constraint that item intercepts are equal across groups. The configural model was evaluated with the same reference values used in the CFA stage. Metric and scalar models were judged invariant when ΔCFI < 0.01 and ΔRMSEA ≤ 0.015 [56, 58]. The Δχ^2^ statistic is reported descriptively; however, it is not used as an invariance criterion because it is sensitive to sample size [59].

Based on the best-fitting CFA model, we proceed with analyses within the IRT framework. Whereas factor analysis assumes a linear relationship between a factor and an item, IRT models a nonlinear relationship between the latent trait and the items, allowing researchers to determine the segment of the trait continuum in which each item provides the most information, improving diagnostic precision [60]. In this phase, we adopted a confirmatory multidimensional graded response model (MGRM), which presumes a theoretically defined multifactor structure and accommodates polytomous items with ordinal responses [61, 62].

The MGRM extends the two-parameter logistic (2PL) model by incorporating multiple dimensions; each item’s parameters are estimated only for the dimension it is intended to measure [63]. Model fit was evaluated with the M2* index, which is tailored to multidimensional IRT models [64]; adequate fit is indicated by a nonsignificant M2* (*p* > 0.05). We also inspected the CFI, TLI, and RMSEA, applying the same reference values used in the CFA stage (CFI and TLI > 0.90 and RMSEA < 0.08 denote acceptable fit [50]. Considering that the use of the CFI, TLI, and RMSEA in the MGRM is still exploratory, these indices should be interpreted cautiously.

The MGRM focused on each item’s discrimination (*a*) and difficulty or thresholds (*b*). The discrimination parameter indicates how well an item differentiates individuals with low versus high levels of the latent trait; higher values signify greater discriminative power. The conventional cutoffs are none (a = 0), very low (0.10–0.34), low (0.35–0.64), moderate (0.65–1.34), high (1.35–1.69), and very high or perfect (≥ 1.70) [65]. The difficulty parameter represents the theta level needed to have at least a 50% probability of endorsing an item in a given category or above [63]. An item’s thresholds equal the number of response categories minus one; thus, each DASS-21 item (four categories, 0–3) has three thresholds (*b₁, b₂, b₃*). Higher *b* values indicate that endorsing the category requires a greater level of the latent trait.

In addition, test information and conditional reliability curves were generated for each subscale and presented separately for the Honduran and Colombian samples. Both sets of curves illustrate measurement precision across the latent trait continuum and indicate the range in which the instrument provides the highest reliability and information [66]. The interpretation of conditional reliability values followed conventional guidelines consistent with those applied in the factor analysis stage, with values ≥ 0.70 considered acceptable and ≥ 0.80 good [67, 68].

Finally, differential item functioning (DIF) by country was evaluated with a likelihood ratio test that used an iterative backward-purification algorithm (scheme = “drop_sequential”) implemented in the *mirt* package [49]. This procedure reduces the risk of misspecified anchors that can mask or inflate DIF in the remaining items [69]. Initially, all item parameters (discrimination and difficulty) were constrained to be equal across countries. Constrained and unconstrained versions of the model were then compared for each item through an iterative anchoring procedure: the item with the largest false discovery rate (FDR) adjusted statistic was freed, the model was re-estimated, and the process was repeated until no additional item showed significant DIF. The final anchor set therefore consisted only of items that remained invariant after purification [33]. Type I errors were controlled with Benjamini–Hochberg correction [70]. *p* values < 0.05 indicated significant cross-cultural DIF.

After the iterative purification procedure was completed, a complementary analysis was performed to obtain test statistics for all items, including those that did not display significant DIF. Specifically, the final anchor set derived from the backward purification algorithm was fixed, and all items were re-tested using a fixed-anchor approach (*scheme* = “drop”) implemented in the *mirt* package. This step provided statistics for each item while preserving the established anchor structure.

For items displaying significant DIF, we inspected whether the differences were uniform (thresholds, parameter *b*) or nonuniform (slopes, parameter *a*) to identify any manifestations of DIF that could bias comparability between groups [33].

## Results

### Confirmatory factor analysis and reliability

The results of the confirmatory factor analysis are presented in Table 2. For the unidimensional and bifactor models, the findings showed good CFI and TLI values (> 0.95); however, the RMSEA indices were unacceptable, exceeding the established cutoff (> 0.08). In contrast, the correlated three-factor and second-order models yielded good CFI and TLI values (> 0.95) and acceptable RMSEA values (< 0.08).

**Table 2:** Fit indices from the confirmatory factor analyses of the tested models

Model	Sample	χ^2^ (df)	CFI	TLI	RMSEA (90% CI)
Unidimensional	Honduras	1254.21 (170)	.990	.988	.076 (.072 –.082)
	Colombia	1612.86 (170)	.992	.991	.086 (.082 –.089)
	Full sample	2747.51 (170)	.990	.989	.082 (.079 –.085)
Correlated three-factor	Honduras	1138.41 (186)	.991	.990	.068 (.065 –.072)
	Colombia	1340.29 (186)	.994	.993	.073 (.070 –.077)
	Full sample	2372.13 (186)	.992	.991	.072 (.069 –.075)
Second order	Honduras	1138.41 (186)	.991	.990	.068 (.065 –.072)
	Colombia	1340.29 (186)	.994	.993	.073 (.070 –.077)
	Full sample	2363.31 (186)	.992	.991	.072 (.069 –.075)
Bifactor	Honduras	5150.08 (169)	.956	.945	.164 (.160 –.168)
	Colombia	5479.78 (169)	.971	.964	.165 (.161 –.168)
	Full sample	10,444.43 (169)	.962	.952	.164 (.161 –.167)

*χ*^*2*^ chi-square statistic, *df* degrees of freedom, *CFI* Comparative fit index, *TLI* Tucker–Lewis index, *RMSEA* Root mean square error of approximation, *CI* Confidence interval

When factor loadings were examined for the models that exhibited acceptable fit, the depression factor had λ values ranging from 0.666 to 0.952 (*p* < 0.01); anxiety, λ = 0.453–0.963 (*p* < 0.01); and stress, λ = 0.650–0.851 (*p* < 0.01). These loadings were comparable in both the correlated three-factor and hierarchical models. In the correlated three-factor model, the interfactor correlations were high: depression–anxiety, *r* = 0.959–0.971 (*p* < 0.01); depression–stress, *r* = 0.864–0.884 (*p* < 0.01); and anxiety–stress, *r* = 0.882–0.921 (*p* < 0.01). In the hierarchical model, the second-order general factor loaded significantly (*p* < 0.01) on the first-order factors of depression (λ = 0.960–0.975) and stress (λ = 0.886–0.922); however, its loading on anxiety was nonsignificant (λ = 0.996–0.999, *p* > 0.01), suggesting that this specific dimension cannot be attributed to the higher-order factor (see Supplementary Tables S3 and S4 for detailed loadings by total sample and by country). Even though the anxiety factor’s loading approaches a perfect correlation with the higher-order factor, the lack of statistical significance may indicate collinearity between the specific and general factors. Given these findings, the correlated three-factor model was identified as the structure that best explains the DASS-21. The reliability estimates for this model are presented in Table 3 and show acceptable values for each factor across samples (Ω and CR > 0.800; AVE > 0.500).

**Table 3:** Reliability of the DASS-21 factors

Factor	Sample	Ω	CR	AVE
Depression	Full sample	.909	.939	.690
	Honduras	.897	.929	.655
	Colombia	.923	.949	.728
Anxiety	Full sample	.835	.880	.521
	Honduras	.845	.892	.547
	Colombia	.829	.871	.504
Stress	Full sample	.877	.907	.583
	Honduras	.876	.907	.583
	Colombia	.880	.910	.590

*Ω* McDonald’s omega, *CR* Composite reliability, *AVE* Average variance extracted

### Measurement invariance

Table 4 reports the results of the factorial invariance analysis between Honduran and Colombian students. The findings demonstrated configural, metric, and scalar invariance for the correlated three-factor model (ΔCFI < 0.01; ΔRMSEA ≤ 0.015), indicating factorial equivalence and permitting valid comparisons between the two samples.

**Table 4:** Configural, metric, and scalar invariance by country

Invariance model	χ^2^(df)	Δχ^2^(Δdf)	CFI	ΔCFI	RMSEA (90% CI)	ΔRMSEA
Configural	2468.70 (372)	-	.993	-	.071 (.068 –.074)	-
Metric	2729.94 (390)	261.24 (18)	.992	**.001**	.073 (.070 –.076)	**.002**
Scalar	2723.28 (429)	254.58 (57)	.992	**.001**	.069 (.066 –.071)	**.002**

Bold values indicate measurement invariance

*χ*^*2*^ chi-square statistic, *df* degrees of freedom, *Δχ*^*2*^ chi-square difference, *CFI* Comparative fit index, *ΔCFI* change in comparative fit index, *RMSEA* Root mean square error of approximation, *CI* Confidence interval, *ΔRMSEA* change in RMSEA

### Multidimensional graded response model

The MGRM showed good fit to the data, M2* (145) = 3,168.492, *p* > 0.05, CFI = 0.951, TLI = 0.943, and RMSEA = 0.075 (90% CI = 0.072–0.078). Table 5 reports the item parameters for each subscale by country. With respect to discrimination (parameter *a*), the depression subscale ranged from 1.225 for DASS_5 (moderate discrimination) to 2.533 for DASS_13 (very high) in the Honduran sample and from 1.499 for DASS_5 (high) to 3.264 for DASS_16 (very high) in the Colombian sample. For the anxiety subscale, the values ranged from 1.020 for DASS_2 (moderate) to 2.321 for DASS_19 (very high) among Honduran students and from 0.890 for DASS_2 (moderate) to 2.737 for DASS_15 (very high) among Colombian students. For the stress subscale, the discrimination values varied between 1.514 for DASS_8 (high) and 3.522 for DASS_12 (very high) in the Honduran sample and between 1.432 for DASS_8 (high) and 3.264 for DASS_12 (very high) in the Colombian sample.

**Table 5:** DASS-21 item parameters estimated with the multidimensional graded response model

Item	Honduras				Colombia			
	*a*	*b1*	*b2*	*b3*	*a*	*b1*	*b2*	*b3*
*Depression*								
Dass_3	1.850	− 0.131	1.276	2.439	2.329	− 0.152	1.301	2.416
Dass_5	1.225	− 0.274	1.327	2.699	1.499	− 0.725	0.900	2.176
Dass_10	2.269	0.108	1.239	2.125	2.946	0.061	1.062	1.881
Dass_13	2.533	− 0.715	0.646	1.510	2.456	− 0.766	0.667	1.643
Dass_16	2.435	0.116	1.344	2.269	3.264	0.270	1.352	2.221
Dass_17	1.845	0.365	1.385	2.034	1.962	0.368	1.433	2.255
Dass_21	1.951	0.571	1.510	2.246	2.367	0.681	1.491	2.180
*Anxiety*								
Dass_2	1.020	1.026	2.576	3.941	0.890	0.479	2.140	3.640
Dass_4	1.608	1.465	2.517	3.318	1.343	1.386	2.820	4.143
Dass_7	2.302	0.998	1.856	2.496	1.808	0.963	2.013	2.872
Dass_9	1.771	0.310	1.397	2.190	1.783	0.132	1.221	2.114
Dass_15	2.317	0.141	1.144	1.827	2.737	0.210	1.056	1.868
Dass_19	2.321	0.322	1.190	1.894	1.846	0.358	1.267	2.126
Dass_20	2.212	0.049	1.118	1.715	2.177	0.210	1.182	2.002
*Stress*								
Dass_1	1.965	− 0.798	0.512	1.555	2.357	− 0.849	0.446	1.611
Dass_6	1.595	− 0.057	1.231	2.098	1.769	− 0.441	0.924	1.992
Dass_8	1.514	0.226	1.367	2.422	1.432	− 0.244	0.900	2.142
Dass_11	2.373	− 0.744	0.423	1.255	2.238	− 0.760	0.425	1.505
Dass_12	3.522	− 0.467	0.497	1.195	3.264	− 0.533	0.468	1.307
Dass_14	2.224	0.065	1.204	2.035	1.922	0.132	1.374	2.393
Dass_18	2.144	− 0.652	0.550	1.293	2.212	− 0.613	0.547	1.519

*a* = discrimination parameter; *b₁, b₂, b₃* = difficulty thresholds for each item response category

Regarding the difficulty parameter, Table 5 shows that the thresholds for all the items follow an ascending pattern, which is consistent with the ordinal nature of the scale’s items. Most depression items—except DASS_3, DASS_5, and DASS_13—displayed positive values in both the Honduran and Colombian samples, suggesting that above-average levels of the latent trait were required to endorse categories *b2* and *b3*. All anxiety items in both samples yielded values greater than zero, indicating that this is the most difficult subscale; participants needed higher latent-trait levels to endorse any category (*b1*, *b2*, or *b3*).

For the stress subscale, every *b1* coefficient was negative (except DASS_8 and DASS_14 in Honduras and DASS_14 in Colombia), making this subscale more efficient at identifying individuals with low or average latent trait levels than the other subscales. Specifically, the most difficult items in the Honduran sample were DASS_5 (depression, *b3* = 2.699), DASS_2 (anxiety, *b3* = 3.941), and DASS_8 (stress, *b3* = 2.422). In the Colombian sample, the most difficult items were DASS_3 (depression, *b3* = 2.416), DASS_4 (anxiety, *b3* = 4.143), and DASS_14 (stress, *b3* = 2.393).

**Fig.2:**
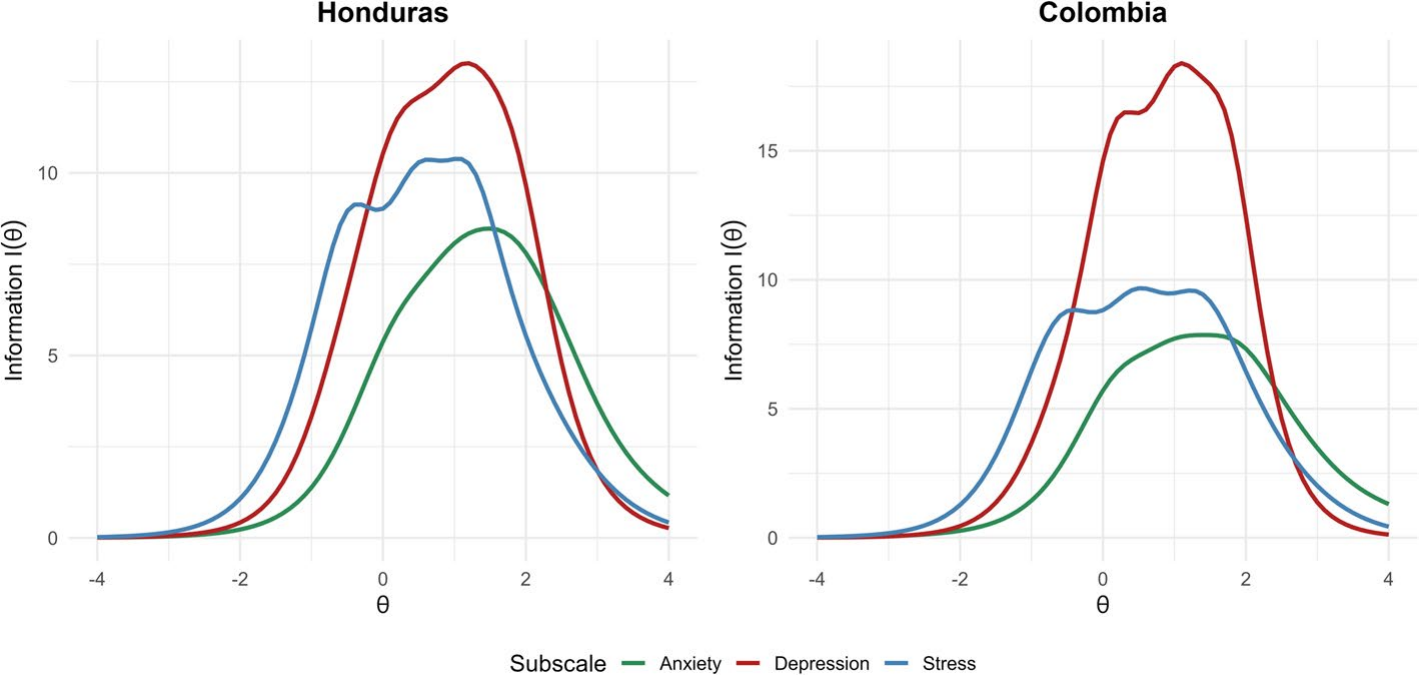
Information curves for the depression, anxiety, and stress subscales by country

Figure 2 shows the information curves for each DASS-21 subscale by country, indicating how much information each dimension provides across different levels of the latent trait. The results reveal that, in both Honduras and Colombia, the depression subscale supplies the greatest amount of information (Honduras I[θ] > 10; Colombia I[θ] > 15). Taken together, the three subscales display acceptable precision and are more suitable for assessing individuals whose latent trait levels are above average (θ > 0).

**Fig. 3:**
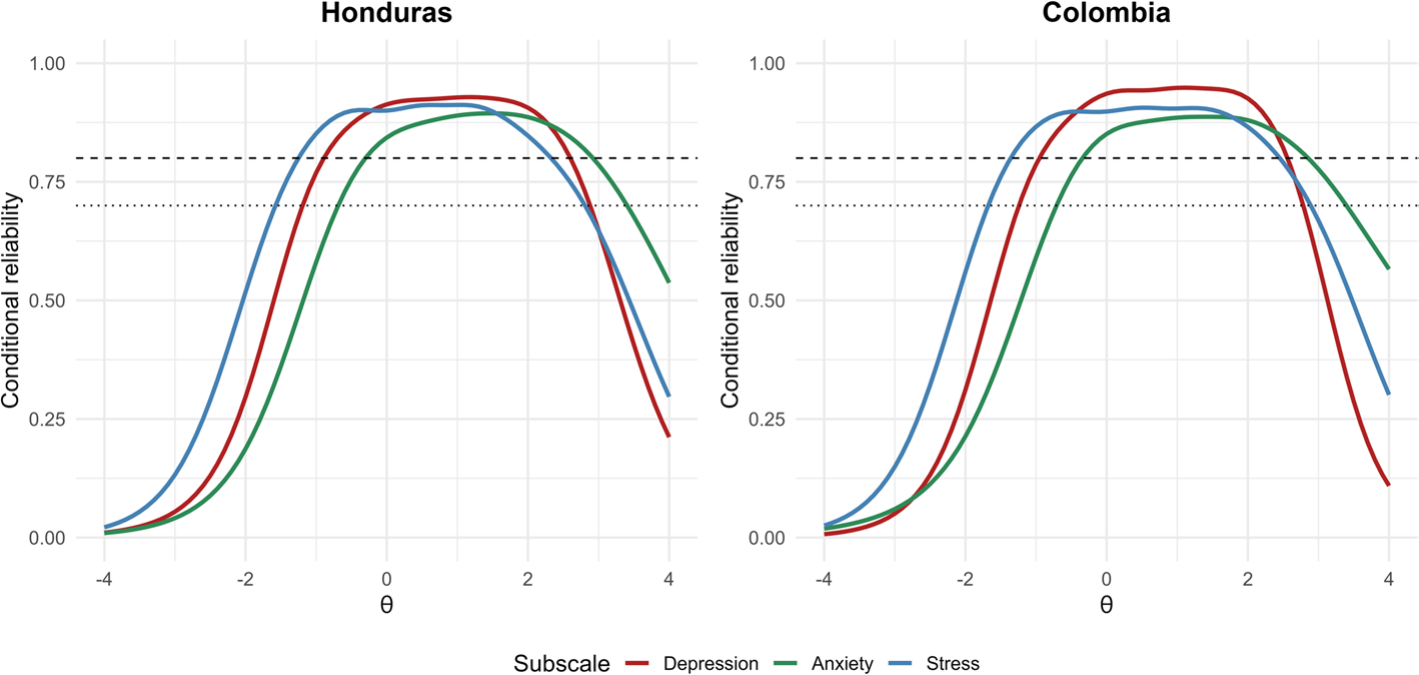
Conditional reliability curves for the depression, anxiety, and stress subscales by country. Note. The lighter dashed line represents reliability values ≥.70, and the darker dashed line represents values ≥.80

Figure 3 depicts the conditional reliability curves for each DASS-21 subscale by country. The results indicate that, in both samples, reliability remained high for latent trait levels roughly between − 1 and 2. Within this interval, all three subscales reach values above 0.70, denoting acceptable precision, and most approach or exceed 0.80, reflecting good reliability. Outside this range—particularly at the lowest and highest ends of the trait continuum—measurement precision decreases.

### Differential item functioning

Following the iterative anchoring procedure, the algorithm halted purification with 17 items remaining invariant (*p* > 0.05). Table 6 lists the items that showed differential item functioning by country (*p* < 0.05). Only items DASS_2 (“I noticed that my mouth was dry”), DASS_5 (“I found it difficult to work up the initiative to do things”), DASS_6 (“I tended to overreact to situations”), and DASS_8 (“I felt that I was using a large amount of nervous energy”) displayed significant DIF. Subsequent analyses indicated that these items exhibited uniform DIF on the difficulty parameter (*b*). The nonuniform DIF (parameter *a*) remains invariant. The DIF test statistics for all DASS-21 items, including those without significant DIF, are reported in Supplementary Table S5.

**Table 6:** Items displaying differential item functioning between Honduran and Colombian students

Item	Dimension	X^2^ (df = 6)	*p*	DIF
Dass_2	Anxiety	38.251	<.001	Uniform
Dass_5	Depression	32.142	<.001	Uniform
Dass_6	Stress	33.570	<.001	Uniform
Dass_8	Stress	39.729	<.001	Uniform

*χ*^*2*^ chi-square test statistic, *df* degrees of freedom, *DIF* Differential item functioning

## Discussion

This research examined the psychometric properties of the DASS-21—dimensionality, reliability, cross-national invariance, item-level discrimination and difficulty parameters, and differential item functioning (DIF)—among Honduran and Colombian university students. The results indicate that the scale displays adequate psychometric qualities in these samples.

With respect to the factorial structure, a comparative analysis of different models identified the correlated three-factor solution as optimal, supporting the tripartite model of depression, anxiety, and stress (CFI and TLI > 0.95; RMSEA < 0.08; λ > 0.40; significant interfactor correlations, *p* < 0.001). Although systematic reviews have reported favorable evidence for unidimensional and bifactor structures [14, 19], the structure identified in Honduras and Colombia aligns with the dimensionality proposed in the original study [44] and with findings from diverse contexts and languages—including Bangladesh [22], the United States [23], China [71], Russia, the United Kingdom, and Poland [18].

The internal-consistency estimates for each factor showed satisfactory omega and composite-reliability values, which is consistent with previous research [22, 71].

The depression, anxiety, and stress factors in the correlated model showed very high associations (*r* > 0.86, *p* < 0.01), supporting the possible presence of a general psychological-distress construct consistent with the tripartite model [16]. In the hierarchical model, however, the anxiety factor’s loading was nonsignificant (*p* > 0.05) despite λ = 0.99—a pattern that likely reflects severe multicollinearity rather than a lack of association. Accordingly, anxiety seems to preserve psychophysiological nuances that are either unique or entirely overlapping with the general factor—subtleties the hierarchical model failed to capture and that call for a more flexible approach.

The bifactor model’s nonacceptable RMSEA (> 0.08) further indicates that the data do not fit a structure in which general and specific factors coexist orthogonally. Future studies in Honduran and Colombian contexts could benefit from alternative specifications such as bifactor exploratory structural equation modeling (bifactor-ESEM), which allows cross-loadings and reduces collinearity [72, 73].

The DASS-21 showed configural, metric, and scalar invariance between Honduran and Colombian students, permitting cross-cultural comparisons of latent means for each dimension. Previous studies have reported partial metric and scalar invariance between Pakistan and Germany [17]; threshold invariance across Poland, Russia, the United Kingdom, and the United States [18]; and scalar invariance of the correlated three-factor model across Canada, Hong Kong, Romania, Taiwan, and the United States [19]. To our knowledge, the current research is the first to analyze invariance evidence for the scale between two Latin American countries.

The item parameters derived from the MGRM indicated that the items furnish adequate information about their underlying latent dimension. Discrimination estimates ranged from moderate to very high across all three subscales in both countries, a pattern consistent with earlier findings [22, 31]. With respect to difficulty, the anxiety items displayed the highest thresholds in both Honduras and Colombia, suggesting that this subscale is calibrated to detect moderate-to-high levels of the latent trait and may not adequately identify individuals with low trait levels. The depression items, although more evenly distributed, still tended to target above-average trait levels. In contrast, the stress dimension yielded the lowest difficulty values, making it more sensitive to respondents at the lower end of the trait continuum. This subscale-specific pattern replicates previous reports, especially in nonclinical samples [22, 74]. Overall, and as illustrated by the information curves, the depression subscale contributed the greatest amount of information in both samples, corroborating results from prior graded response model studies [22, 31, 74].

In addition, the conditional reliability curves derived from the IRT analysis indicated acceptable reliability across the middle range of the latent trait (approximately − 1 < θ < 2), with values generally exceeding 0.70. Outside this interval, particularly at the lower and upper extremes, measurement precision decreased, as expected in instruments calibrated for nonclinical populations. This pattern suggests that the DASS-21 provides consistent and precise estimates of depression, anxiety, and stress for individuals with average to moderately elevated symptom levels.

Although the scale was invariant at the construct level across countries, the MGRM-based DIF analysis revealed that four items exhibited uniform DIF (statistically significant differences in item difficulty thresholds). These differences may reflect cultural variation in how the symptoms assessed by those items are perceived. Differences in how emotional distress is expressed or labeled in everyday language may influence how respondents from Honduras and Colombia interpret and respond to specific items. For instance, subtle linguistic nuances or culturally specific connotations associated with terms related to depression, anxiety, and stress could partly account for the observed DIF. This hypothesis, however, could be explored further through validity studies based on response-process evidence, which examine the cognitive processes underlying item responses [75].

From a practical perspective, understanding these cultural and linguistic sources of DIF is essential to ensure the comparability of item responses when making cross-group comparisons at the symptom level. In contrast, the DIF analysis indicated that all item discrimination parameters remained invariant, suggesting that the items still differentiate equally well across countries among individuals with varying levels of the latent trait. Nevertheless, cross-cultural comparisons of these specific items should be made with caution.

### Implications and limitations

The findings of this study have important implications for assessing mental health among Honduran and Colombian university students. First, the DASS-21 can serve as a valid and reliable screening tool for depression, anxiety, and stress. Moreover, item-level interpretation of those items with high discrimination and difficulty values can guide the identification of clinically relevant symptomatology and the subsequent implementation of more effective interventions. Second, evidence of measurement invariance enables comparative analyses that can enhance the monitoring of mental health indicators in these Latin American regions.

Although this study makes valuable contributions, several limitations must be acknowledged. First, in both Honduras and Colombia, participants were selected through nonprobability convenience sampling, which restricts the generalizability of the findings to the higher-education populations of those settings. Nevertheless, in each country, the survey link was widely circulated through institutional channels, yielding a heterogeneous sample in terms of sociodemographic and academic characteristics, as shown in Table 1.

Second, the current research included only higher-education students. Additional studies in Honduras and Colombia should incorporate samples with different educational levels and age groups, as well as clinical and nonclinical populations, to expand the potential applicability of the DASS-21 across diverse sociodemographic and clinical profiles.

Third, measurement invariance and DIF analyses were conducted only at the country level. To strengthen context-specific evidence, future studies should extend these analyses by incorporating contrast variables such as sex, age, field of study, and other clinically relevant features. Given this study’s cross-sectional design, additional longitudinal research is needed to examine DASS-21 invariance and DIF across repeated measurements.

Fourth, although the present work evaluated several psychometric properties of the DASS-21, further evidence of its validity through complementary psychometric approaches is essential. For example, studies that establish validity via relationships with external variables or that adopt contemporary paradigms such as network analysis, which is epistemologically distinct from latent-variable theory and centers on the interconnections among the assessed symptoms (items), are needed.

Finally, the present study did not estimate the magnitude and direction of DIF effects. Although Table 5 provides item parameter estimates for Honduras and Colombia that may serve as a reference for qualitative interpretation, there is currently no standardized or widely adopted procedure for reporting DIF effect sizes in multidimensional graded response models. We therefore acknowledge this as a methodological limitation and highlight it as a relevant direction for future research.

## Conclusion

The findings of this study provide favorable psychometric evidence for the use of the DASS-21 as a mental-health screening tool that assesses a correlated tripartite model—including depression, anxiety, and stress—among Honduran and Colombian university students. The DASS-21 is therefore a valuable instrument that higher-education institutions can deploy to allocate mental health services and interventions more efficiently.

The measurement invariance between Honduran and Colombian students supports cross-cultural comparisons of latent means in both countries, although individual‒item comparisons warrant caution for items that exhibit DIF. Cross-national research can foster multicenter studies and strengthen the epidemiological surveillance of mental health indicators among university students in these Latin American contexts.

## Supplementary Information

Supplementary Material 1.

Supplementary Material 2.

## Data Availability

The datasets used and analyzed during the current study are available from the corresponding author on reasonable request.

## Abbreviations

AVE: Average variance extracted
CFA: Confirmatory factor analysis
CFI: Comparative fit index
CR: Composite reliability
DASS-21: Depression, Anxiety and Stress Scale- 21
DIF: Differential item function
HDI: Human Development Index
IRT: Item response theory
MGCFA: Multigroup confirmatory factor analysis
MGRM: Multidimensional graded response model
RMSEA: Root mean square error of approximation
TLI: Tucker–Lewis index
