# Supplementary material for: Dimensionality, reliability, invariance, and item analysis of the depression, anxiety, and stress scale-21 (DASS-21) in Honduran and Colombian university students
Source: BMC Psychol. 2025 Nov 25;13:1413. doi: 10.1186/s40359-025-03742-5 (PMC12751876; doi:10.1186/s40359-025-03742-5)
Supplement: Supplementary file 1 — Supplementary Material 1. [file 40359_2025_3742_MOESM1_ESM.docx]

**Supplementary material 1- Tables**

Dimensionality, reliability, invariance, and item analysis of the Depression, Anxiety, and Stress Scale-21 (DASS-21) in Honduran and Colombian university students

**Table S1. Descriptive statistics for variables used in this study**

| **Items** | **Full Sample** | | | | **Honduras** | | | | **Colombia** | | | |
| --- | --- | --- | --- | --- | --- | --- | --- | --- | --- | --- | --- | --- |
|  | **M** | **SD** | **Skew** | **Kurt** | **M** | **SD** | **Skew** | **Kurt** | **M** | **SD** | **Skew** | **Kurt** |
| **Depression** |  |  |  |  |  |  |  |  |  |  |  |  |
| Dass_3 | 0.78 | 0.88 | 0.93 | 0.02 | 0.78 | 0.90 | 0.95 | 0.01 | 0.78 | 0.87 | 0.91 | 0.02 |
| Dass_5 | 0.97 | 0.96 | 0.67 | -0.56 | 0.86 | 0.94 | 0.82 | -0.35 | 1.07 | 0.97 | 0.55 | -0.70 |
| Dass_10 | 0.75 | 0.96 | 1.06 | 0.01 | 0.71 | 0.93 | 1.13 | 0.26 | 0.79 | 0.99 | 0.99 | -0.22 |
| Dass_13 | 1.15 | 1.00 | 0.51 | -0.79 | 1.14 | 1.01 | 0.53 | -0.79 | 1.16 | 0.99 | 0.49 | -0.80 |
| Dass_16 | 0.66 | 0.88 | 1.22 | 0.56 | 0.68 | 0.89 | 1.17 | 0.48 | 0.63 | 0.88 | 1.26 | 0.62 |
| Dass_17 | 0.66 | 0.96 | 1.31 | 0.53 | 0.67 | 0.98 | 1.31 | 0.48 | 0.65 | 0.93 | 1.29 | 0.55 |
| Dass_21 | 0.55 | 0.91 | 1.56 | 1.25 | 0.57 | 0.91 | 1.52 | 1.17 | 0.54 | 0.91 | 1.58 | 1.31 |
| **Anxiety** |  |  |  |  |  |  |  |  |  |  |  |  |
| Dass_2 | 0.52 | 0.82 | 1.53 | 1.50 | 0.42 | 0.74 | 1.84 | 2.75 | 0.62 | 0.87 | 1.28 | 0.68 |
| Dass_4 | 0.24 | 0.57 | 2.73 | 7.73 | 0.22 | 0.57 | 2.98 | 9.11 | 0.25 | 0.56 | 2.47 | 6.29 |
| Dass_7 | 0.32 | 0.68 | 2.32 | 5.02 | 0.30 | 0.68 | 2.50 | 5.88 | 0.34 | 0.69 | 2.15 | 4.25 |
| Dass_9 | 0.67 | 0.91 | 1.23 | 0.52 | 0.62 | 0.89 | 1.36 | 0.90 | 0.72 | 0.92 | 1.11 | 0.20 |
| Dass_15 | 0.70 | 0.93 | 1.14 | 0.24 | 0.70 | 0.93 | 1.18 | 0.36 | 0.70 | 0.93 | 1.11 | 0.11 |
| Dass_19 | 0.63 | 0.92 | 1.31 | 0.61 | 0.62 | 0.91 | 1.35 | 0.74 | 0.65 | 0.92 | 1.26 | 0.46 |
| Dass_20 | 0.71 | 0.93 | 1.16 | 0.32 | 0.75 | 0.96 | 1.13 | 0.23 | 0.68 | 0.91 | 1.18 | 0.37 |
| **Stress** |  |  |  |  |  |  |  |  |  |  |  |  |
| Dass_1 | 1.20 | 0.95 | 0.37 | -0.77 | 1.19 | 0.97 | 0.4 | -0.81 | 1.2 | 0.92 | 0.34 | -0.74 |
| Dass_6 | 0.86 | 0.93 | 0.86 | -0.21 | 0.78 | 0.93 | 1.03 | 0.11 | 0.95 | 0.93 | 0.70 | -0.42 |
| Dass_8 | 0.79 | 0.95 | 0.94 | -0.21 | 0.66 | 0.9 | 1.19 | 0.41 | 0.90 | 0.98 | 0.74 | -0.61 |
| Dass_11 | 1.22 | 0.99 | 0.37 | -0.91 | 1.24 | 1.02 | 0.38 | -0.96 | 1.20 | 0.97 | 0.36 | -0.88 |
| Dass_12 | 1.13 | 1.02 | 0.49 | -0.88 | 1.12 | 1.03 | 0.52 | -0.88 | 1.14 | 1.01 | 0.46 | -0.89 |
| Dass_14 | 0.67 | 0.85 | 1.16 | 0.54 | 0.70 | 0.88 | 1.13 | 0.43 | 0.64 | 0.83 | 1.17 | 0.62 |
| Dass_18 | 1.15 | 1.01 | 0.48 | -0.85 | 1.18 | 1.03 | 0.48 | -0.91 | 1.13 | 0.99 | 0.48 | -0.82 |
| Note. M = Mean; SD = Standard deviation; Skew = Skewness; Kurt = Kurtosis. All items were scored on a 0–3 scale; therefore, the minimum value is 0 and the maximum value is 3 for all items. | | | | | | | | | | | | |

**Table S2. Response category percentages of DASS-21 scale items**

| **Items** | **Full sample** | | | | **Honduras** | | | | **Colombia** | | | |
| --- | --- | --- | --- | --- | --- | --- | --- | --- | --- | --- | --- | --- |
|  | **0** | **1** | **2** | **3** | **0** | **1** | **2** | **3** | **0** | **1** | **2** | **3** |
| **Depression** | |  |  |  |  |  |  |  |  |  |  |  |
| Dass_3 | 46.70 | 34.10 | 13.60 | 5.60 | 47.39 | 33.09 | 13.40 | 6.12 | 45.99 | 35.12 | 13.81 | 5.09 |
| Dass_5 | 38.70 | 34.70 | 17.60 | 9.00 | 44.51 | 32.19 | 15.65 | 7.64 | 33.13 | 37.10 | 19.5 | 10.27 |
| Dass_10 | 53.10 | 26.50 | 12.20 | 8.10 | 54.05 | 27.61 | 11.15 | 7.19 | 52.20 | 25.45 | 13.29 | 9.06 |
| Dass_13 | 30.20 | 38.40 | 18.00 | 13.50 | 30.76 | 38.22 | 17.27 | 13.76 | 29.59 | 38.48 | 18.72 | 13.2 |
| Dass_16 | 56.40 | 27.10 | 10.80 | 5.60 | 54.37 | 29.04 | 10.73 | 5.86 | 58.41 | 25.28 | 10.87 | 5.44 |
| Dass_17 | 60.30 | 22.10 | 9.20 | 8.50 | 60.70 | 21.49 | 8.18 | 9.62 | 59.97 | 22.61 | 10.09 | 7.33 |
| Dass_21 | 67.10 | 17.60 | 8.30 | 7.00 | 65.89 | 18.81 | 8.19 | 7.11 | 68.25 | 16.48 | 8.46 | 6.82 |
| **Anxiety** |  |  |  |  |  |  |  |  |  |  |  |  |
| Dass_2 | 64.70 | 22.40 | 8.80 | 4.10 | 70.81 | 19.55 | 6.76 | 2.88 | 58.84 | 25.19 | 10.7 | 5.26 |
| Dass_4 | 82.30 | 13.10 | 3.30 | 1.30 | 84.35 | 10.97 | 3.06 | 1.62 | 80.41 | 15.1 | 3.54 | 0.95 |
| Dass_7 | 77.60 | 15.10 | 4.60 | 2.60 | 79.77 | 13.40 | 4.05 | 2.79 | 75.58 | 16.82 | 5.18 | 2.42 |
| Dass_9 | 56.70 | 26.40 | 10.40 | 6.60 | 59.68 | 25.20 | 8.91 | 6.21 | 53.75 | 27.52 | 11.82 | 6.90 |
| Dass_15 | 55.60 | 25.70 | 11.90 | 6.90 | 54.73 | 27.54 | 10.26 | 7.47 | 56.34 | 23.90 | 13.46 | 6.30 |
| Dass_19 | 60.50 | 22.30 | 10.50 | 6.70 | 60.99 | 22.61 | 9.64 | 6.76 | 59.97 | 22.09 | 11.39 | 6.56 |
| Dass_20 | 54.30 | 27.70 | 10.40 | 7.70 | 52.21 | 29.52 | 9.18 | 9.09 | 56.26 | 25.97 | 11.48 | 6.30 |
| **Stress** |  |  |  |  |  |  |  |  |  |  |  |  |
| Dass_1 | 26.00 | 39.30 | 24.00 | 10.70 | 27.07 | 38.49 | 22.57 | 11.87 | 24.94 | 40.03 | 25.28 | 9.75 |
| Dass_6 | 43.50 | 34.60 | 14.10 | 7.80 | 49.19 | 31.74 | 11.33 | 7.73 | 37.96 | 37.36 | 16.74 | 7.94 |
| Dass_8 | 50.60 | 27.30 | 14.90 | 7.20 | 56.74 | 25.81 | 11.69 | 5.76 | 44.69 | 28.73 | 18.03 | 8.54 |
| Dass_11 | 27.40 | 36.60 | 22.40 | 13.50 | 27.30 | 36.67 | 20.72 | 15.32 | 27.52 | 36.58 | 24.07 | 11.82 |
| Dass_12 | 32.70 | 34.70 | 19.40 | 13.20 | 33.45 | 34.71 | 18.08 | 13.76 | 32.01 | 34.69 | 20.71 | 12.60 |
| Dass_14 | 53.50 | 30.80 | 10.80 | 4.80 | 52.34 | 31.08 | 10.90 | 5.68 | 54.70 | 30.46 | 10.79 | 4.06 |
| Dass_18 | 30.80 | 36.60 | 19.20 | 13.50 | 30.06 | 36.99 | 17.64 | 15.30 | 31.49 | 36.15 | 20.62 | 11.73 |
| Note. 0 = Never; 1 = Sometimes; 2 = A lot of the time; 3 = Most or all the time. All values are expressed as percentages. | | | | | | | | | | | | |

**Table S3. Factorial loadings and correlations of the three correlated factors of the DASS-21**

| **Factor** | **Samples** | | |
| --- | --- | --- | --- |
|  | **Full sample** | **Honduras** | **Colombia** |
| **Depression** |  |  |  |
| Dass_3 | .805* | .775* | .837* |
| Dass_5 | .693* | .666* | .727* |
| Dass_10 | .912* | .876* | .952* |
| Dass_13 | .862* | .859* | .865* |
| Dass_16 | .895* | .871* | .918* |
| Dass_17 | .803* | .789* | .813* |
| Dass_21 | .825* | .809* | .841* |
| **Anxiety** |  |  |  |
| Dass_2 | .487* | .563* | .453* |
| Dass_4 | .599* | .655* | .549* |
| Dass_7 | .708* | .738* | .681* |
| Dass_9 | .710* | .717* | .706* |
| Dass_15 | .963* | .937* | .991* |
| Dass_19 | .730* | .760* | .703* |
| Dass_20 | .764* | .773* | .762* |
| **Stress** |  |  |  |
| Dass_1 | .751* | .720* | .784* |
| Dass_6 | .715* | .712* | .728* |
| Dass_8 | .655* | .671* | .650* |
| Dass_11 | .767* | .774* | .763* |
| Dass_12 | .851* | .849* | .852* |
| Dass_14 | .798* | .810* | .787* |
| Dass_18 | .791* | .792* | .796* |
| **Correlations** |  |  |  |
| Depression ↔ Anxiety | .966* | .959* | .971* |
| Depression ↔ Stress | .874* | .884* | .864* |
| Anxiety ↔ Stress | .903* | .921* | .882* |
| * = *p* < 0.01 |  |  |  |

**Table S4. Factorial loadings of the second-order model of the DASS-21**

| **Factor** | **Samples** | | |
| --- | --- | --- | --- |
|  | **Full sample** | **Honduras** | **Colombia** |
| **Depression** |  |  |  |
| Dass_3 | .805* | .775* | .837* |
| Dass_5 | .693* | .666* | .727* |
| Dass_10 | .912* | .876* | .952* |
| Dass_13 | .862* | .859* | .865* |
| Dass_16 | .895* | .871* | .918* |
| Dass_17 | .803* | .789* | .813* |
| Dass_21 | .825* | .809* | .841* |
| **Anxiety** |  |  |  |
| Dass_2 | .487* | .563* | .453* |
| Dass_4 | .599* | .655* | .549* |
| Dass_7 | .708* | .738* | .681* |
| Dass_9 | .710* | .717* | .706* |
| Dass_15 | .963* | .937* | .991* |
| Dass_19 | .730* | .760* | .703* |
| Dass_20 | .764* | .773* | .762* |
| **Stress** |  |  |  |
| Dass_1 | .751* | .720* | .784* |
| Dass_6 | .715* | .712* | .728* |
| Dass_8 | .655* | .671* | .650* |
| Dass_11 | .767* | .774* | .763* |
| Dass_12 | .851* | .849* | .852* |
| Dass_14 | .798* | .810* | .787* |
| Dass_18 | .791* | .792* | .796* |
| **General factor** |  |  |  |
| Depression | .967* | .960* | .975* |
| Anxiety | .999 | .999 | .996 |
| Stress | .904* | .922* | .886* |
| * = *p* < 0.01 |  |  |  |

**Table S5. Differential item functioning (DIF) results for the DASS-21 items by country (Colombia vs. Honduras)**

| **Item** | **Dimension** | **X^2^ (df = 3)** | **p** |
| --- | --- | --- | --- |
| DASS_3 | Depression | 2.055 | .620 |
| DASS_5 | Depression | 32.142 | **< .001** |
| DASS_10 | Depression | 5.912 | .187 |
| DASS_13 | Depression | 1.064 | .786 |
| DASS_16 | Depression | 4.619 | .283 |
| DASS_17 | Depression | 6.160 | .182 |
| DASS_21 | Depression | 2.305 | .597 |
| DASS_2 | Anxiety | 38.251 | **< .001** |
| DASS_4 | Anxiety | 10.916 | .053 |
| DASS_7 | Anxiety | 7.568 | .107 |
| DASS_9 | Anxiety | 9.638 | .064 |
| DASS_15 | Anxiety | 9.408 | .064 |
| DASS_19 | Anxiety | 1.907 | .622 |
| DASS_20 | Anxiety | 13.109 | .190 |
| DASS_1 | Stress | 5.445 | .213 |
| DASS_6 | Stress | 33.570 | **< .001** |
| DASS_8 | Stress | 39.729 | **< .001** |
| DASS_11 | Stress | 7.987 | .097 |
| DASS_12 | Stress | 2.971 | .489 |
| DASS_14 | Stress | 3.657 | .395 |
| DASS_18 | Stress | 8.434 | .088 |
| Note: χ² = chi-square statistic; df = degrees of freedom. | | | |
