# Supplementary material for: Dimensionality, reliability, invariance, and item analysis of the depression, anxiety, and stress scale-21 (DASS-21) in Honduran and Colombian university students
Source: BMC Psychol. 2025 Nov 25;13:1413. doi: 10.1186/s40359-025-03742-5 (PMC12751876; doi:10.1186/s40359-025-03742-5)
Supplement: Supplementary file 2 — Supplementary Material 2. [file 40359_2025_3742_MOESM2_ESM.docx]

**Supplementary material 2- Syntax**

Dimensionality, reliability, invariance, and item analysis of the Depression, Anxiety, and Stress Scale-21 (DASS-21) in Honduran and Colombian university students

############################################

# DASS-21 (Honduras & Colombia): Supplementary R Syntax

# R 4.4.2 | lavaan 0.6-16 | semTools 0.5-6 | mirt 1.45.1

############################################

# --- Packages ---

library(readxl)

library(dplyr)

library(psych)

library(lavaan)

library(semPlot)

library(semTools)

library(mirt)

# --- Data import ---

# # Replace the path below with the directory where the dataset is stored

Ruta_Excel <- "path_to_dataset/DASS21_HND_COL.xlsx"

DASS21_HND_COL <- read_excel(Ruta_Excel)

# --- Split by country (for descriptive/auxiliary fits) ---

# Note: 'País' is the grouping variable with levels "Honduras" and "Colombia".

DASS21_HND <- DASS21_HND_COL %>% filter(País == "Honduras")

DASS21_COL <- DASS21_HND_COL %>% filter(País == "Colombia")

############################################

# PHASE 1: FACTOR MODELING (CFA + RELIABILITY + INVARIANCE)

############################################

# --- CFA Model 1: Unidimensional ---

# Note: Ordered categorical indicators; WLSMV estimator.

Modelo_DASS21_1 <- '

DASS21 =~ DASS_1 + DASS_2 + DASS_3 + DASS_4 + DASS_5 + DASS_7 +

DASS_8 + DASS_9 + DASS_10 + DASS_11 + DASS_12 + DASS_13 +

DASS_14 + DASS_15 + DASS_16 + DASS_17 + DASS_18 + DASS_19 +

DASS_20 + DASS_21

'

fitModelo_DASS21_1 <- sem(Modelo_DASS21_1, data = DASS21_HND_COL, ordered = TRUE, estimator = "WLSMV")

fitMeasures(fitModelo_DASS21_1, fit.measures = c("chisq","df","cfi","tli","rmsea","rmsea.ci.lower","rmsea.ci.upper"))

reliability(fitModelo_DASS21_1)

# Per-country fits (descriptive only)

fitModelo_DASS21_11 <- sem(Modelo_DASS21_1, data = DASS21_HND, ordered = TRUE, estimator = "WLSMV")

fitMeasures(fitModelo_DASS21_11, fit.measures = c("chisq","df","cfi","tli","rmsea","rmsea.ci.lower","rmsea.ci.upper"))

reliability(fitModelo_DASS21_11)

fitModelo_DASS21_12 <- sem(Modelo_DASS21_1, data = DASS21_COL, ordered = TRUE, estimator = "WLSMV")

fitMeasures(fitModelo_DASS21_12, fit.measures = c("chisq","df","cfi","tli","rmsea","rmsea.ci.lower","rmsea.ci.upper"))

reliability(fitModelo_DASS21_12)

# --- CFA Model 2: Three correlated factors (Dep, Ans, Est) ---

# Note: This is the target/best-fitting structure.

Modelo_DASS21_2 <- '

Dep =~ DASS_3 + DASS_5 + DASS_10 + DASS_13 + DASS_16 + DASS_17 + DASS_21

Ans =~ DASS_2 + DASS_4 + DASS_7 + DASS_9 + DASS_15 + DASS_19 + DASS_20

Est =~ DASS_1 + DASS_6 + DASS_8 + DASS_11 + DASS_12 + DASS_14 + DASS_18

'

fitModelo_DASS21_2 <- sem(Modelo_DASS21_2, data = DASS21_HND_COL, ordered = TRUE, estimator = "WLSMV")

fitMeasures(fitModelo_DASS21_2, fit.measures = c("chisq","df","cfi","tli","rmsea","rmsea.ci.lower","rmsea.ci.upper"))

summary(fitModelo_DASS21_2, fit.measures = TRUE, standardized = TRUE)

reliability(fitModelo_DASS21_2)

# Per-country fits (descriptive only)

fitModelo_DASS21_21 <- sem(Modelo_DASS21_2, data = DASS21_HND, ordered = TRUE, estimator = "WLSMV")

fitMeasures(fitModelo_DASS21_21, fit.measures = c("chisq","df","cfi","tli","rmsea","rmsea.ci.lower","rmsea.ci.upper"))

summary(fitModelo_DASS21_21, fit.measures = TRUE, standardized = TRUE)

reliability(fitModelo_DASS21_21)

fitModelo_DASS21_22 <- sem(Modelo_DASS21_2, data = DASS21_COL, ordered = TRUE, estimator = "WLSMV")

fitMeasures(fitModelo_DASS21_22, fit.measures = c("chisq","df","cfi","tli","rmsea","rmsea.ci.lower","rmsea.ci.upper"))

summary(fitModelo_DASS21_22, fit.measures = TRUE, standardized = TRUE)

reliability(fitModelo_DASS21_22)

# --- Composite Reliability (from standardized loadings) ---

# Note: Convenience function to compute a CR-like index from standardized λ.

composite_reliability <- function(x, reduce = TRUE, cutoff = 0.3) {

# x: vector of standardized loadings for one factor

cargas <- abs(x)

if (isTRUE(reduce)) cargas <- cargas[cargas > cutoff]

e <- 1 - cargas^2

cr <- (sum(cargas))^2 / ((sum(cargas))^2 + sum(e))

return(cr)

}

# CR for the three correlated-factor model (overall and by country)

cargas_DASS21_2 <- inspect(fitModelo_DASS21_2, "std")$lambda

apply(cargas_DASS21_2, 2, composite_reliability)

cargas_DASS21_21 <- inspect(fitModelo_DASS21_21, "std")$lambda

apply(cargas_DASS21_21, 2, composite_reliability)

cargas_DASS21_22 <- inspect(fitModelo_DASS21_22, "std")$lambda

apply(cargas_DASS21_22, 2, composite_reliability)

# (Optional) CR for unidimensional model using the first column of λ

composite_reliability(cargas_DASS21_2[,1])

# --- CFA Model 3: Second-order factor (G over Dep, Ans, Est) ---

Modelo_DASS21_3 <- '

Dep =~ DASS_3 + DASS_5 + DASS_10 + DASS_13 + DASS_16 + DASS_17 + DASS_21

Ans =~ DASS_2 + DASS_4 + DASS_7 + DASS_9 + DASS_15 + DASS_19 + DASS_20

Est =~ DASS_1 + DASS_6 + DASS_8 + DASS_11 + DASS_12 + DASS_14 + DASS_18

G =~ Dep + Ans + Est

'

fitModelo_DASS21_3 <- sem(Modelo_DASS21_3, data = DASS21_HND_COL, ordered = TRUE, estimator = "WLSMV", std.lv = TRUE)

fitMeasures(fitModelo_DASS21_3, fit.measures = c("chisq","df","cfi","tli","rmsea","rmsea.ci.lower","rmsea.ci.upper"))

summary(fitModelo_DASS21_3, fit.measures = TRUE, standardized = TRUE)

reliability(fitModelo_DASS21_3)

# Per-country fits (descriptive only)

fitModelo_DASS21_31 <- sem(Modelo_DASS21_3, data = DASS21_HND, ordered = TRUE, estimator = "WLSMV", std.lv = TRUE)

fitMeasures(fitModelo_DASS21_31, fit.measures = c("chisq","df","cfi","tli","rmsea","rmsea.ci.lower","rmsea.ci.upper"))

summary(fitModelo_DASS21_31, fit.measures = TRUE, standardized = TRUE)

reliability(fitModelo_DASS21_31)

fitModelo_DASS21_32 <- sem(Modelo_DASS21_3, data = DASS21_COL, ordered = TRUE, estimator = "WLSMV", std.lv = TRUE)

fitMeasures(fitModelo_DASS21_32, fit.measures = c("chisq","df","cfi","tli","rmsea","rmsea.ci.lower","rmsea.ci.upper"))

summary(fitModelo_DASS21_32, fit.measures = TRUE, standardized = TRUE)

reliability(fitModelo_DASS21_32)

# --- CFA Model 4: Bifactor (G + three specific factors; orthogonal specifics) ---

Modelo_DASS21_4 <- '

Dep =~ DASS_3 + DASS_5 + DASS_10 + DASS_13 + DASS_16 + DASS_17 + DASS_21

Ans =~ DASS_2 + DASS_4 + DASS_7 + DASS_9 + DASS_15 + DASS_19 + DASS_20

Est =~ DASS_1 + DASS_6 + DASS_8 + DASS_11 + DASS_12 + DASS_14 + DASS_18

G =~ DASS_1 + DASS_2 + DASS_3 + DASS_4 + DASS_5 + DASS_7 + DASS_8 +

DASS_9 + DASS_10 + DASS_11 + DASS_12 + DASS_13 + DASS_14 +

DASS_15 + DASS_16 + DASS_17 + DASS_18 + DASS_19 + DASS_20 + DASS_21

'

fitModelo_DASS21_4 <- sem(Modelo_DASS21_4, data = DASS21_HND_COL, ordered = TRUE,

orthogonal = TRUE, estimator = "WLSMV", std.lv = TRUE)

fitMeasures(fitModelo_DASS21_4, fit.measures = c("chisq","df","cfi","tli","rmsea","rmsea.ci.lower","rmsea.ci.upper"))

summary(fitModelo_DASS21_4, fit.measures = TRUE, standardized = TRUE)

reliability(fitModelo_DASS21_4)

# Per-country fits (descriptive only)

fitModelo_DASS21_41 <- sem(Modelo_DASS21_4, data = DASS21_HND, ordered = TRUE,

orthogonal = TRUE, estimator = "WLSMV", std.lv = TRUE)

fitMeasures(fitModelo_DASS21_41, fit.measures = c("chisq","df","cfi","tli","rmsea","rmsea.ci.lower","rmsea.ci.upper"))

summary(fitModelo_DASS21_41, fit.measures = TRUE, standardized = TRUE)

reliability(fitModelo_DASS21_41)

fitModelo_DASS21_42 <- sem(Modelo_DASS21_4, data = DASS21_COL, ordered = TRUE,

orthogonal = TRUE, estimator = "WLSMV", std.lv = TRUE)

fitMeasures(fitModelo_DASS21_42, fit.measures = c("chisq","df","cfi","tli","rmsea","rmsea.ci.lower","rmsea.ci.upper"))

summary(fitModelo_DASS21_42, fit.measures = TRUE, standardized = TRUE)

reliability(fitModelo_DASS21_42)

###########################################################################

# --- Measurement invariance by country (three correlated-factor model) ---

# Note: Group variable is 'País'; thresholds are constrained at scalar level.

Configural_DASS21_3f <- cfa(Modelo_DASS21_2, data = DASS21_HND_COL, group = "País",

ordered = TRUE, estimator = "WLSMV")

fitMeasures(Configural_DASS21_3f, c("chisq","df","cfi","tli","rmsea","rmsea.ci.lower","rmsea.ci.upper"))

Metric_DASS21_3f <- cfa(Modelo_DASS21_2, data = DASS21_HND_COL, group = "País",

group.equal = "loadings", ordered = TRUE, estimator = "WLSMV")

fitMeasures(Metric_DASS21_3f, c("chisq","df","cfi","tli","rmsea","rmsea.ci.lower","rmsea.ci.upper"))

Scalar_DASS21_3f <- cfa(Modelo_DASS21_2, data = DASS21_HND_COL, group = "País",

group.equal = c("loadings","thresholds"),

ordered = TRUE, estimator = "WLSMV")

fitMeasures(Scalar_DASS21_3f, c("chisq","df","cfi","tli","rmsea","rmsea.ci.lower","rmsea.ci.upper"))

############################################

# PHASE 2: IRT (MGRM) AND DIF

############################################

# --- IRT data prep for mirt ---

# Note: Build IRT dataset with country and item columns; remove missing responses if needed.

Dass21_TRI <- DASS21_HND_COL %>%

select(País, starts_with("DASS_"))

# Country-specific subsets (for descriptive MGRM fits)

Dass21_TRI_honduras <- Dass21_TRI %>% filter(País == "Honduras")

Dass21_TRI_colombia <- Dass21_TRI %>% filter(País == "Colombia")

# Optional complete-case version (used if M2/DIF require no missing)

Dass21_TRI_semNA <- Dass21_TRI %>% tidyr::drop_na(starts_with("DASS_"))

# --- MGRM structure (three correlated dimensions) ---

# Note: Correlated 3D graded response model.

sintaxe_3f <- "

DEP = DASS_3, DASS_5, DASS_10, DASS_13, DASS_16, DASS_17, DASS_21

ANS = DASS_2, DASS_4, DASS_7, DASS_9, DASS_15, DASS_19, DASS_20

EST = DASS_1, DASS_6, DASS_8, DASS_11, DASS_12, DASS_14, DASS_18

COV = DEP*EST*ANS

"

# --- MGRM fit (both countries pooled) ---

# Note: MHRM is recommended for 3+ dimensions with polytomous items; SE=TRUE for parameter SEs.

mod_conf <- mirt(

data = Dass21_TRI[-1], # remove country col for item matrix

model = sintaxe_3f,

itemtype = "graded",

SE = TRUE,

method = "MHRM"

)

summary(mod_conf, suppress = 0.1) # brief parameter summary

coef(mod_conf, simplify = TRUE, IRTpars = TRUE) # discrimination (a) and thresholds (b)

M2(mod_conf) # global fit index for IRT model

# --- MGRM fits by country (descriptive parameter inspection) ---

mod_conf_HND <- mirt(

data = Dass21_TRI_honduras[-1],

model = sintaxe_3f,

itemtype = "graded",

SE = TRUE,

method = "MHRM"

)

summary(mod_conf_HND, suppress = 0.1)

coef(mod_conf_HND, simplify = TRUE, IRTpars = TRUE)

mod_conf_COL <- mirt(

data = Dass21_TRI_colombia[-1],

model = sintaxe_3f,

itemtype = "graded",

SE = TRUE,

method = "MHRM"

)

summary(mod_conf_COL, suppress = 0.1)

coef(mod_conf_COL, simplify = TRUE, IRTpars = TRUE)

###########################################################################

# --- Multigroup MGRM for DIF (country as grouping factor) ---

# Note: Start with all items invariant (as anchors) except means/variances free; then apply DIF search.

mg_3f <- multipleGroup(

data = Dass21_TRI[-1],

model = sintaxe_3f,

group = Dass21_TRI$País,

itemtype = "graded",

invariance = c("free_mean","free_var", colnames(Dass21_TRI[-1])),

method = "MHRM",

SE = TRUE

)

print(mg_3f)

# --- DIF search (backward purification; FDR-adjusted) ---

# Note: 'drop_sequential' iteratively frees parameters for the item with largest adj. statistic until no DIF remains.

result_DIF <- DIF(

mg_3f,

which.par = c("a1","a2","a3","d1","d2","d3"),

scheme = "drop_sequential",

p.adjust = "fdr"

)

print(result_DIF)

summary(result_DIF, type = "parameters") # lists items/parameters flagged

# Optional: Focused summaries for specific items

summary(result_DIF, which.items = c("DASS_2","DASS_5","DASS_6","DASS_8"))

# --- Fixed-anchor DIF re-test (using purified anchor set) ---

# Note: Refit DIF with a fixed set of anchors from the purification phase (confirmatory DIF check).

anchor_final <- result_DIF$anchor

result_DIF_all <- DIF(

mg_3f,

which.par = c("a1","a2","a3","d1","d2","d3"),

scheme = "drop",

anchor = anchor_final,

p.adjust = "fdr"

)

print(result_DIF_all)

summary(result_DIF_all, type = "parameters")

# --- Uniform vs Non-uniform DIF (parameter-focused tests) ---

# Note: Non-uniform DIF is probed via discrimination parameters (a1–a3); uniform DIF via thresholds (d1–d3).

# Non-uniform DIF (a parameters)

result_DIFa <- DIF(

mg_3f,

which.par = c("a1","a2","a3"),

scheme = "drop_sequential",

p.adjust = "fdr"

)

print(result_DIFa)

summary(result_DIFa, type = "parameters")

# Uniform DIF (d parameters)

result_DIFb <- DIF(

mg_3f,

which.par = c("d1","d2","d3"),

scheme = "drop_sequential",

p.adjust = "fdr"

)

print(result_DIFb)

summary(result_DIFb, type = "parameters")
